# Soluble intercellular adhesion molecule-1 is a prognostic marker in colorectal carcinoma

**DOI:** 10.1007/s00384-018-3198-0

**Published:** 2018-11-23

**Authors:** Vera S. Schellerer, Melanie C. Langheinrich, Veronika Zver, Robert Grützmann, Michael Stürzl, Olaf Gefeller, Elisabeth Naschberger, Susanne Merkel

**Affiliations:** 1 Department of Surgery, University Medical Center Erlangen, Friedrich-Alexander-University Erlangen-Nürnberg, Krankenhausstr. 12, 91054 Erlangen, Germany; 2 Division of Molecular and Experimental Surgery, Department of Surgery, University Medical Center Erlangen, Friedrich-Alexander-University Erlangen-Nürnberg, Erlangen, Germany; 3 Department of Medical Informatics, Biometry and Epidemiology, Friedrich-Alexander- University of Erlangen-Nürnberg, Erlangen, Germany

**Keywords:** Colorectal carcinoma, CEA, sICAM-1, Survival

## Abstract

**Purpose:**

Serological tumor markers are routinely used to monitor tumor onset and progression. In colorectal carcinoma (CRC), the carcinoembryonic antigen (CEA) is roughly elevated in 50% of patients at initial diagnosis. Soluble ICAM-1 (sICAM-1) is elevated in different cancers. The aim of this study was to evaluate the prognostic relevance of sICAM-1 combined with CEA in patients with CRC.

**Methods:**

In blood samples of 297 CRC patients, sICAM-1 was determined by ELISA and CEA by microparticle enzyme immunoassay the day before oncologic resection. Separation in patients with sICAM-1^high^ and sICAM-1^low^ was performed by minimum *p* value approach; separation in CEA normal and elevated was performed according to the established diagnostic cutoff. Clinical data were obtained from the prospective collected data from the Erlangen Registry for Colorectal Carcinomas.

**Results:**

Cancer-related 5-year survival rate of patients with sICAM-1^low^ (< 290 ng/ml, *n* = 208) was significantly increased (83.4%) as compared to that of patients with sICAM-1^high^ (≥ 290 ng/ml, *n* = 89) (66.2%; *p* < 0.001). Patients with normal CEA concentrations (*n* = 199; 90.8%) showed a significantly (*p* < 0.001) improved cancer-related 5-year survival rate compared to patients with elevated CEA concentrations (*n* = 98; 52.1%). Moreover, high sICAM-1 was an independent risk factor (hazard ratio 1.6) in multivariate analysis. Of note, increased sICAM-1 levels, either within normal or within elevated CEA, allowed to identify high-risk subgroups, both for overall (*p *< 0.001) and cancer-related survival (*p* < 0.001).

**Conclusion:**

Application of a novel risk score combining CEA/sICAM-1 serum concentrations allows the identification of high-risk groups for poor survival in CRC patients.

## Introduction

Colorectal carcinoma (CRC) is among the leading cancers and responsible for 1.4 million new cancer cases and nearly 700,000 deaths per year worldwide [1]. CRC occurs predominantly in industrialized countries; thus, the highest incidence rates are found in Europe and North America [1]. The overall 5-year survival rate is approximately 65% [2].

Serological tumor markers indicating recurrence of CRC would be a useful and simple tool during follow-up. At present, the most accepted serological tumor markers are increased concentrations of the carcinoembryonic antigen (CEA). In healthy individuals, CEA serum concentrations are ranging from < 2.5 to 5.0 ng/ml [3]. However, at the time of initial diagnosis of CRC, only 39–50% of patients present with elevated CEA concentrations [4–7]. Patients with preoperative CEA serum levels within the normal range (< 5 ng/ml) showed a significant better prognosis than patients with elevated levels [8]. Several studies confirmed the clinical usefulness of CEA to determine prognosis and detect recurrence during follow-up. Accordingly, CEA is well integrated in clinical routine processes [9–14].

Intercellular adhesion molecule-1 (ICAM-1, CD54) is a member of the immunoglobulin superfamily. Several cells, such as fibroblasts, endothelial cells, or epithelial cells, express ICAM-1 as a surface glycoprotein, consisting of five extracellular domains, a transmembrane domain, and a short cytoplasmatic domain [15–18]. In tumor-associated fibroblasts, the cellular expression of ICAM-1 is increased compared to normal tissue-associated fibroblasts, indicating a tumor microenvironment–dependent upregulation of its expression [19]. A soluble form of ICAM-1 (sICAM-1) is present in elevated concentrations in the sera of different kinds of cancers, for example, gastric cancer, pancreatic cancer, melanoma, and CRC [20–27]. A positive correlation of sICAM-1 serum concentrations and tumor stage in CRC was described for example by Mantur et al. in 40 patients, by Basouglu et al. in 67 patients, and by Kang et al. in 56 patients [21, 28–31]. These patient cohorts were generally very small and follow-up of the patients was at the most 3 months after surgery.

At present, it is unclear how sICAM-1 is generated. In one study, shedding of the membrane-bound ICAM-1 from cell surfaces by tumor necrosis factor-α-converting enzyme (TACE/ADAM-17) was suggested as a potential mechanism [32]. In agreement with the small size of the transmembrane and cytoplasmic domains, soluble ICAM-1 is only slightly smaller in size than its membrane-bound form. Its ability to bind lymphocyte function-associated antigen (LFA-1) is still preserved but exhibits different affinities depending on the form of the molecule: monomeric sICAM-1 and truncated sICAM-1 (domains 1 and 2) exhibit low affinity to LFA-1, whereas a dimerized form of immobilized sICAM-1 binds with a high affinity to LFA-1 [18]. Accordingly, sICAM-1 binds to circulating cytotoxic lymphocytes which might block immune recognition of tumor cells and may facilitate tumor progression [33]. Moreover, sICAM-1 has the ability to promote angiogenesis by the stimulation of neovascularization in the chick CAM assay [34].

The aim of this study was to evaluate the potential impact of serum concentration of sICAM-1 as a prognostic marker in combination with routinely determined CEA at the time of initial diagnosis of CRC in a large patient cohort (UICC I-IV) with a substantial follow-up period.

## Patients and methods

### Patients

Serum sICAM-1 concentrations and clinical outcomes were analyzed in 297 patients with an invasive CRC treated at our institution between September 2005 and September 2013. Patients receiving tumor resection with systematic lymph node dissection (complete mesocolic excision/total mesorectal excision) without pretreatment were included in this study only. All data were collected prospectively and patients were followed until death or January 1, 2017. Routine follow-up was carried out at 6-months intervals for the initial 2 years and yearly thereafter for a total period of 5 years [5]. After completion of regular follow-up, patients or their family doctors were contacted at longer intervals. No patients were lost during follow-up. Stages were determined according to the eighth edition of the Tumor Node Metastasis (TNM) classification of the Union Internationale Contre le Cancer (UICC) [35]. The current classification defines stages I and II as carcinomas without metastases, whereas stage III is defined by regional lymph node metastases and stage IV by distant metastases. R classification was defined according to the proposal by UICC [35]: R0, no residual tumor; R1, microscopic residual tumor; R2, macroscopic residual tumor; and RX, presence of residual tumor cannot be assessed. Controls included patients entering the ambulance of the Medical Center due to other non-malignant diseases. These controls were confirmed to have no malignant disease at the time of blood draw. The study was approved by the local ethics committee and all included patients signed an informed consent.

### Measurement of sICAM-1 concentrations by ELISA

Blood samples were collected by peripheral venous puncture the day before surgery during routine preoperative blood draw. Soluble ICAM-1 and CEA levels were determined in samples from the same blood draw. Blood was drawn in S-Monovette 9 ml, Clotting Activator/Serum (Sarstedt, Nümbrecht, Germany, ref. 02.1063) and was allowed to clot for 30 min. After centrifugation at 2500×*g* for 10 min at room temperature, the supernatant was collected in aliquots and frozen at − 80 °C until analysis. Soluble ICAM-1 was measured using the human ICAM-1/CD54 non-allele-specific Quantikine ELISA kit (Cat. no. SCIM00, R&D Systems, Inc. Minneapolis, USA) following the manufacturer’s instructions. Every blood sample was measured in duplicates. The mean value of sICAM-1 was calculated and used for further analysis.

### Measurement of CEA concentrations by microparticle enzyme immunoassay

CEA serum concentrations were determined during routine preoperative blood draw the day before surgery. A microparticle enzyme immunoassay (ARCHITECT CEA Reagent Kit; ref. 7K68-27; Abbott; Wiesbaden) was used according to the manufacturer’s instructions. CEA concentrations of 5 ng/ml and higher were categorized as elevated and below 5 ng/ml as normal according to the manufacturer’s reference.

### Statistical analysis

Statistical analysis was performed using the statistical software Statistical Package for Social Sciences (SPSS) for Windows (version 21.0, SPSS Inc., Chicago, IL, USA). A *p* value < 0.05 was considered significant, and a *p* value < 0.005 as highly significant. After testing for normal distribution (Kolmogorov-Smirnov test), variables are expressed as mean ± standard deviation (SD) and median and were compared by the Kruskal-Wallis test. Survival curves were determined by the Kaplan-Meier method and differences in survival were compared by logrank test. The 95% confidence intervals (95% CI) were calculated according to Greenwood [36]. Cox regression analysis was used for multivariate analyses. For identification of independent prognostic factors, all variables with a *p* < 0.05 in univariate analysis were included into the multivariate model. For the analysis of overall survival, death of any cause was defined as an event. For the analysis of cancer-related survival, a cancer-related death was defined as an event, i.e., death with (recurred) locoregional carcinoma and/or distant metastases. A minimum *p* value approach was used to derive an optimal discrimination of the total patient group into two subgroups with different survival prognoses depending on the level of sICAM-1. Using this approach, logrank tests for all different cutoffs of sICAM-1 were performed to find the optimal cutoff level with the lowest *p* value. Following this approach, patients with sICAM-1 concentrations < 290 ng/ml were categorized as sICAM-1^low^, and patients with sICAM-1 concentrations of ≥ 290 ng/ml as sICAM-1^high^.

## Results

**Fig. 1:**
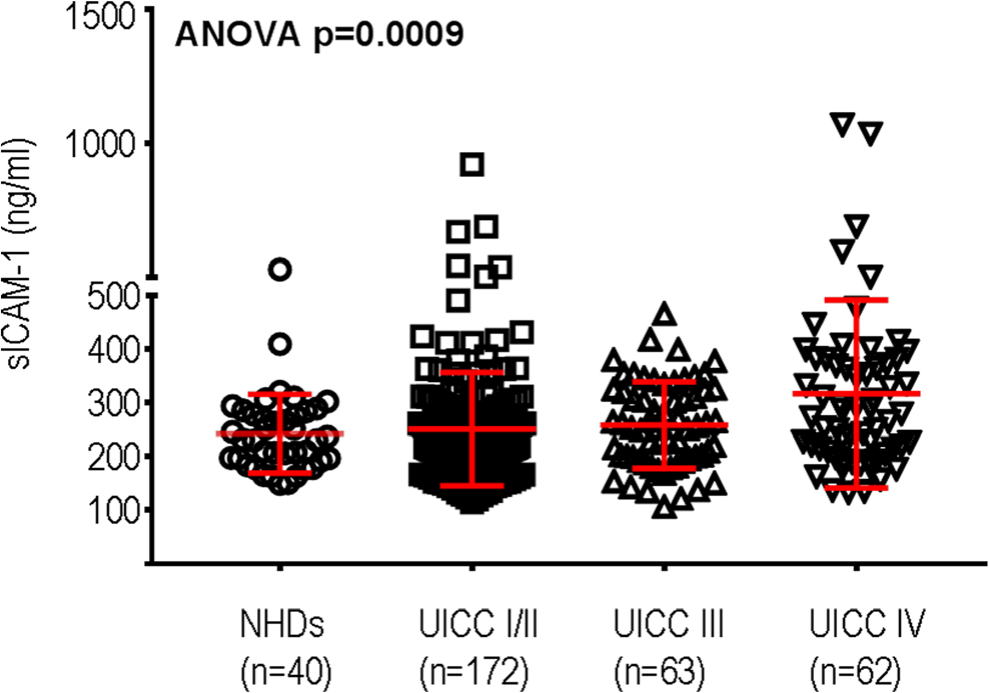
Soluble ICAM-1 concentrations increase significantly (ANOVA, *p* = 0.0009) from healthy donors to patients without lymph node metastasis (UICC I/II) to patients with lymph node metastases and distant metastases in CRC

The aim of this study was to evaluate the prognostic relevance of sICAM-1 serum concentrations at the time of initial diagnosis of CRC in comparison to the routine tumor marker CEA. The study group consisted of 297 patients, including 185 males (62.3%) and 112 females (37.7%); the median age was 67 years (range 22–91 years). The median follow-up was 66 months (range 0.5–141 months). In the study, 61 patients with UICC stage I, 111 patients with UICC stage II, 63 patients with UICC stage III, and 62 patients with UICC stage IV CRC were included (Table 1). The patients’ characteristics, UICC stages, and residual tumor classification (R classification) are summarized in Table 1. The sICAM-1 serum concentrations of all patients varied between a minimum of 106.14 ng/ml and a maximum of 1070.74 ng/ml. The minimum *p* value approach was used to differentiate the patients into groups of high or low sICAM-1 serum levels. Thereby, a cutoff point between 289.48 and 290.55 ng/ml for sICAM was identified (*p* = 0.000023), and the patients were accordingly separated in sICAM^low^ with levels < 290 ng/ml and in sICAM^high^ with ≥ 290 ng/ml. Overall, 208 patients (70.0%) presented with sICAM-1 concentrations below 290 ng/ml (sICAM-1^low^) and 89 patients (30.0%) with sICAM-1 concentrations of 290 ng/ml or higher (sICAM-1^high^; Table 1). The mean sICAM-1 concentrations were 248.5 ng/ml (median 227.8 ng/ml; SD 125.2) for UICC stage I, 252.6 ng/ml (median 234.4 ng/ml; SD 93.1) for UICC stage II, 258.7 ng/ml (median 257.2 ng/ml; SD 80.4) for UICC stage III, and 316.9 ng/ml (median 276.9 ng/ml; SD 175.1) for UICC stage IV (Table 2). In order to compare the overall tumor serum concentrations to healthy people, a cohort of 40 healthy donors (NHDs) was included in this study (Table 1). In the NHDs, the sICAM-1 concentrations varied between a minimum of 149.7 ng/ml and a maximum of 530.62 ng/ml (mean 242.66 ng/ml; median 230.39 ng/ml) (Fig. 1).

**Table 1:** Patients with colorectal carcinoma (*n* = 297) and healthy donors (NHDs, *n* = 40) included in the study

	Patients, *n* = 297	NHDs, *n* = 40
Gender		
Males	185 (62.3%)	19 (50%)^#^
Females	112 (37.7%)	19 (50%)^#^
Age		
Median	67 years	32 years
Range	22–91 years	22–51 years
sICAM-1		
< 290 ng/ml (sICAM-1^low^)	208 (70.0%)	33 (83%)
≥ 290 ng/ml (sICAM-1^high^)	89 (30.0%)	7 (18%)
CEA		
Normal (< 5 ng/ml)	199 (67.0%)	
Elevated (≥ 5 ng/ml)	98 (33.0%)	
UICC stages		
Stage I	61 (20.5%)	
Stage II	111 (37.4%)	
Stage III	63 (21.2%)	
Stage IV	62 (20.9%)	
R classification		
R0	246 (82.8%)	
R1	4 (1.3%)	
R2	40 (13.5%)	
RX	7 (2.4%)	
Localization		
Colon	231 (77.8)	
Rectum	66 (22.2)	

*sICAM-1* soluble intercellular adhesion molecule-1, *CEA* carcinoembryonic antigen, *UICC* Union Internationale Contre le Cancer, *R classification* residual tumor classification

^#^Gender of two donors was not registered

**Table 2:** Soluble ICAM-1 and CEA serum concentrations increase stage dependently in human colorectal carcinoma (*n* = 297)

		sICAM-1 (ng/ml)	*p* value	CEA normal (< 5 ng/ml)	CEA elevated (≥ 5 ng/ml)	*p* value
	*n*	Mean ± SD (median)		*n* (%)	*n* (%)	
All patients	297	266.5 ± 121.5 (241.4)		199	98	
UICC stages						
Stage I + II	172	251.1 ± 105.2 (233.6)		136 (68.3)	36 (37)	
Stage III	63	258.7 ± 80.4 (257.2)		47 (23.6)	16 (16)	
Stage IV	62	316.9 ± 175.1 (276.9)	0.005	16 (8.0)	46 (47)	< 0.001
Male	185	263.5 ± 111.5 (243.8)		119 (59.8)	66 (67)	
Female	112	271.4 ± 136.8 (238.2)	0.897	80 (40.2)	32 (33)	0.207
Age < 67 years	146	270.1 ± 139.9 (243.5)		101 (50.8)	45 (46)	
Age ≥ 67 years	151	263.0 ± 100.9 (239.9)	0.873	98 (49.2)	53 (54)	0.433
CEA normal	199	255.5 ± 106.3 (235.9)				
CEA elevated	98	288.7 ± 145.6 (252.0)	0.045			

*Soluble ICAM-1* soluble intercellular adhesion molecule-1, *CEA* carcinoembryonic antigen, *UICC* Union Internationale Contre le Cancer

### Serum concentrations of sICAM-1 and CEA positively correlate with advanced CRC

A significant increase of sICAM-1 concentrations was detected in advanced CRC. Patients without lymph node metastases (UICC stages I and II) had significant lower sICAM-1 concentrations compared to patients with lymph node metastases (UICC stage III) or distant metastases (UICC stage IV) (*p* = 0.005, Kruskal-Wallis test; Table 2, Fig. 1). Moreover, a significant difference of sICAM-1 concentrations was detected when compared to NHDs (*p* = 0.0009, Fig. 1). Patients with elevated CEA concentrations were more frequent in advanced UICC stages of CRC (*p* < 0.001). Gender and age of patients did not influence sICAM-1 nor CEA concentrations (sICAM-1: *p* = 0.897 for gender, *p* = 0.873 for age; CEA: *p* = 0.207 for gender, *p* = 0.433 for age; Table 2). In addition, patients with elevated CEA concentrations (*n* = 98) presented with significant higher mean concentrations of sICAM-1 (mean 288.7 ng/ml) than patients with normal CEA concentrations (*n* = 199; mean 255.5 ng/ml; *p* = 0.045; Table 2).

### Elevated sICAM-1 levels are associated with a significantly decreased overall and cancer-related survival and serve as independent prognostic factor

**Fig. 2:**
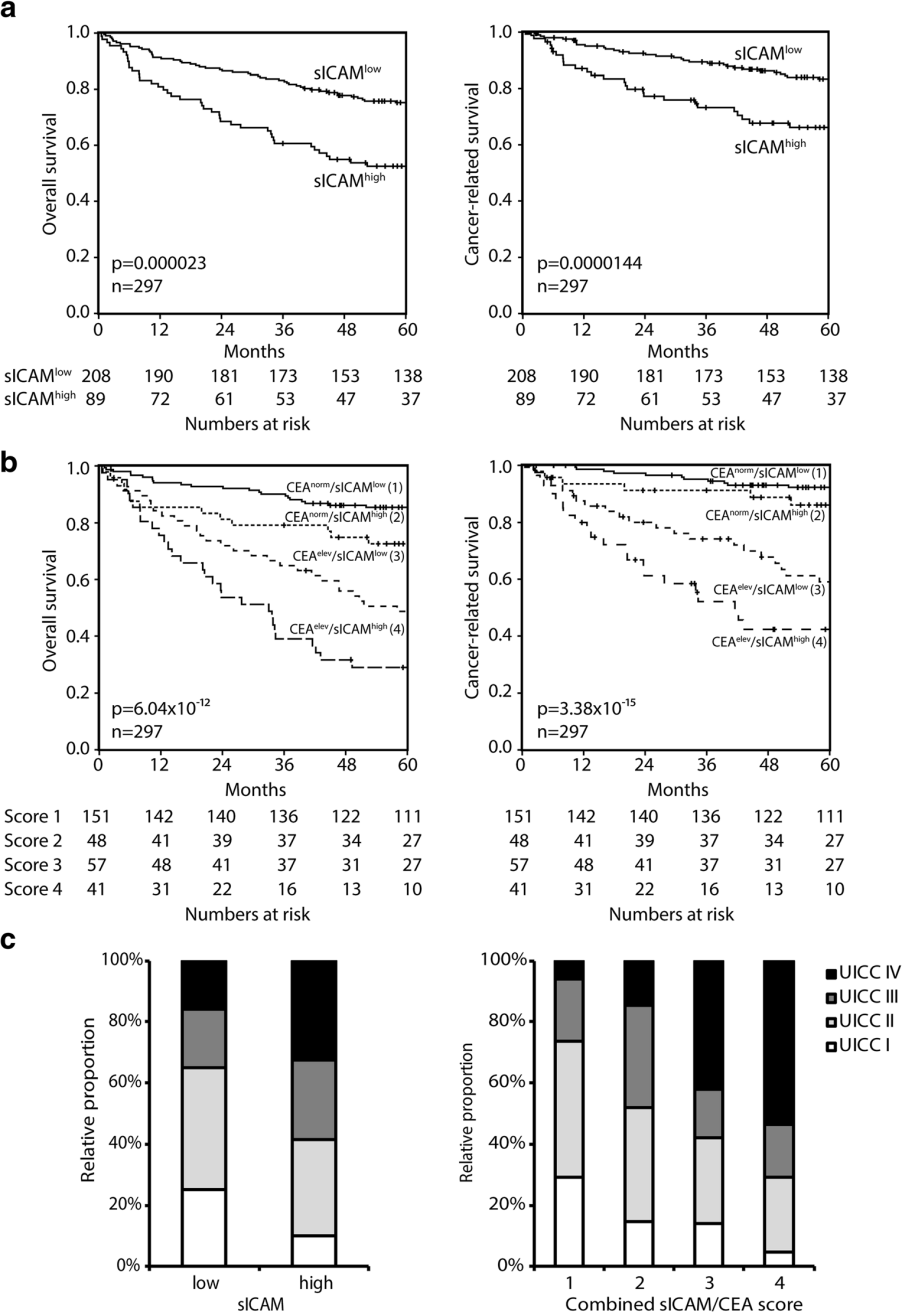
**a** Patients with high sICAM-1 concentrations present with a worse overall (*p* < 0.001) and cancer-related survival (*p* < 0.001) compared to patients with low sICAM-1 concentrations. **b** Combination of CEA and sICAM-1 serum concentrations identifies four significant (*p* < 0.001) different risk groups in CRC patients (*n* = 297). **c** Relative proportion in percent of UICC I–IV stages in patients with low and high sICAM-1 concentrations (left). Relative proportion in percent of UICC I–IV stages of combined sICAM-1/CEA score (right)

Patients with sICAM-1^low^ concentrations displayed a highly significant improved 5-year overall survival compared to patients with sICAM-1^high^ concentrations (75.2% vs. 52.5%, *p* < 0.001; Table 3, Fig. 2a, left panel). Accordingly, also a prolonged cancer-related survival was detected (83.4% vs. 66.2%, *p* < 0.001; Table 3, Fig. 2a, right panel). Moreover, the overall and cancer-related survival was significantly increased in patients with normal CEA concentrations compared to elevated CEA concentrations (overall: CEA normal 82.3% vs. CEA elevated 40.5%; *p* < 0.001; cancer-related: CEA normal 90.8% vs. CEA elevated 52.1%; *p* < 0.001; Table 3). For both parameters, sICAM-1 and CEA, each increased concentrations were associated with decreased overall survival in all stages, but significance was proven for CEA in UICC stage I + II (*p* = 0.009) and for sICAM-1 in UICC stage III (*p* = 0.048; Table 3) only. In a multivariate analysis, elevated levels of sICAM-1 were identified as an independent prognostic factor with an increased hazard ratio of 1.6 (*p* = 0.016; Table 4). Elevated CEA levels were presenting with an increased hazard ratio of 1.5 in the same analysis, but were not significant (*p* = 0.084; Table 4).

**Table 3:** Low soluble ICAM-1 and normal CEA concentrations are associated with improved survival in human colorectal carcinoma (*n* = 297)

		*n*	5-year overall survival (%)/95% CI	*p* value	5-year cancer-related survival (%)/95% CI	*p* value
sICAM-1^low^ (< 290 ng/ml)		208	75.2/69.3–81.1		83.4/78.1–88.7	
sICAM-1^high^ (≥ 290 ng/ml)		89	52.5/42.1–62.9	0.000023	66.2/55.6–76.8	0.0000144
CEA normal (< 5 ng/ml)		199	82.3/77.0–87.6		90.8/86.7–94.9	
CEA elevated (≥ 5 ng/ml)		98	40.5/30.7–50.3	4.123E−10	52.1/41.3–61.9	5.135E−14
UICC stages						
Stage I + II	sICAM-1^low^	135	85.0/78.9–91.1		94.2/90.1–98.3	
	sICAM-1^high^	37	72.6/58.1–87.1	0.097	96.2/85.6–100	0.861
Stage III	sICAM-1^low^	40	82.1/10.1–94.1		94.2/86.4–100	
	sICAM-1^high^	23	64.7/44.9–84.5	0.048	84.1/67.6–100	0.114
Stage IV	sICAM-1^low^	33	27.3/12.0–42.6		28.2/12.5–43.9	
	sICAM-1^high^	29	17.2/3.5–30.9	0.257	19.2/4.5–33.9	0.265
Stage I + II	CEA normal	136	88.2/82.7–93.7		96.0/92.5–99.5	
	CEA elevated	36	60.4/44.1–76.7	0.009	87.9/75.0–100	0.107
Stage III	CEA normal	47	78.1/66.1–90.1		92.3/83.9–100	
	CEA elevated	16	68.8/46.1–91.5	0.990	86.5/69.1–100	0.657
Stage IV	CEA normal	16	43.8/19.5–68.1		43.8/19.5–68.1	
	CEA elevated	46	15.2/4.8–25.6	0.082	16.5/5.3–27.7	0.111

*sICAM-1* soluble intercellular adhesion molecule-1, *CEA* carcinoembryonic antigen, *UICC* Union Internationale Contre le Cancer

All R classifications are included in the survival analysis

**Table 4:** Univariate and multivariate overall survival analysis

		Univariate analysis			Multivariate analysis		
	*n*	5-year survival (%)	95% CI	*p*	Hazard ratio (HR)	95% CI	*p*
sICAM-1^low^	208	75.2	69.3–81.1		1.0		
sICAM-1^high^	89	52.5	42.1–62.9	0.000023	1.6	1.1–2.4	0.016
CEA^normal^	199	82.3	77.0–87.6		1.0		
CEA^elevated^	98	40.5	30.7–50.3	4.123E−10	1.5	1.0–2.2	0.084
Gender							
Male	185	63.0	55.9–70.1		1.0		
Female	112	77.4	69.6–85.2	0.003	0.6	0.4–0.9	0.008
Age							
< 67 years	146	68.9	61.3–76.5				
≥ 67 years	151	68.0	60.6–75.4	0.171			
Localization							
Rectum	66	68.4	56.8–80.0				
Colon	231	68.3	62.2–74.4	0.352			
UICC stages							
Stage I	61	85.0	76.0–94.0		1.0		
Stage II	111	80.9	73.6–88.2		0.8	0.4–1.4	0.347
Stage III	63	75.8	65.0–86.6		1.0	0.5–1.8	0.948
Stage IV	62	22.6	12.2–33.0	< 0.001	1.0	0.4–2.5	0.965
R classification							
R0	246	80.2	75.1–85.3		1.0		
R1	4	25.0	0–67.5		6.6	1.9–23.3	0.003
R2	40	2.5	0–7.4		9.5	3.9–23.1	< 0.001
RX	7	57.1	20.4–93.8	< 0.001	1.9	0.5–7.3	0.356

*sICAM-1* soluble intercellular adhesion molecule-1, *CEA* carcinoembryonic antigen, *UICC* Union Internationale Contre le Cancer, *R classification* residual tumor classification

### High sICAM-1 serum concentrations identify high-risk subgroups within patients with both, normal or elevated CEA concentrations for overall and cancer-related survival

By combination of both markers, a novel score with four subcategories was developed: (1) normal CEA and sICAM-1^low^, (2) normal CEA and sICAM-1^high^, (3) elevated CEA and sICAM-1^low^, and (4) elevated CEA and sICAM-1^high^ allowing the identification of high-risk subgroups (Table 5, Fig. 2b).

**Table 5:** High soluble ICAM-1 concentrations identify a high-risk subgroup within colorectal carcinoma patients presenting with either normal or elevated CEA concentrations

		*n*	5-year survival/95% CI	*p* value
Overall survival				6.037E−12
Score 1	CEA normal, sICAM-1^low^	151	85.4/79.7–91.1	0.025*
Score 2	CEA normal, sICAM-1^high^	48	72.6/59.9–85.3	0.093**
Score 3	CEA elevated, sICAM-1^low^	57	48.7/35.6–61.8	0.017***
Score 4	CEA elevated, sICAM-1^high^	41	29.1/15.2–43.0	
Cancer-related survival				3.382E−15
Score 1	CEA normal, sICAM-1^low^	151	92.2/87.9–96.5	0.155*
Score 2	CEA normal, sICAM-1^high^	48	86.0/75.6–96.4	0.009**
Score 3	CEA elevated, sICAM-1^low^	57	59.1/45.4–72.8	0.028***
Score 4	CEA elevated, sICAM-1^high^	41	42.4/25.9–58.9	

*Soluble ICAM-1* soluble intercellular adhesion molecule-1, *CEA* carcinoembryonic antigen

*Score 1 vs. score 2

**Score 2 vs. score 3

***Score 3 vs. score 4

Considering the overall survival, patients with normal CEA and sICAM-1^low^ serum concentrations had a 5-year survival rate of 85.4% (score 1). Patients with normal CEA and sICAM-1^high^ serum concentrations presented with a significantly decreased 5-year survival of 72.6% (*p* = 0.025, score 2), and patients with elevated CEA and sICAM-1^low^ concentrations with 48.7% (score 3). Patients with elevated CEA and sICAM-1^high^ concentrations presented with the worst 5-year overall survival rates of 29.1% (score 4). The 5-year overall survival rates were significantly different between all four groups (*p* < 0.001) (Table 5, Fig. 2b, left panel).

In patients with normal CEA concentrations, there was only a marginal difference in cancer-related survival between sICAM-1^low^ and sICAM-1^high^ (*p* = 0.155). However, patients with elevated CEA concentrations and sICAM-1^high^ concentrations presented with a significant worse survival compared to patients with elevated CEA and sICAM-1^low^ (42.4% vs. 59.1%; *p* = 0.028; Table 5; Fig. 2b, right panel). The 5-year cancer-related survival rates were significantly different between all four groups (*p* < 0.001) (Table 5; Fig. 2b, right panel).

Importantly, all four UICC stages were represented in both subgroups of patients with low and high sICAM-1 concentrations. Stage IV patients were a fraction of only 32.6% in sICAM-1^high^ patients (Fig. 2c, left). The relative proportion of lower UICC stages decreased in patients with sICAM-1^high^ concentrations. For the combined sICAM-1/CEA score, similar results were found: again all four UICC stages are represented within the four different sICAM-1/CEA scores, but the relative proportion of lower UICC stages also decreases with elevating risk groups. Stage IV patients were a fraction of 42.1% in the combined score 3 and 53.7% in score 4 (Fig. 2c, right). The multivariate analyses revealed that UICC staging even lost its role as an independent prognostic factor when sex, sICAM-1, CEA, and R classification are simultaneously incorporated in the analysis.

## Discussion

CRC is responsible for a large number of cancer diagnoses and deaths per year worldwide. Depending on the pathological UICC stages, further adjuvant therapy is started and prognosis is conceivable. Furthermore, subsequent to operation, a structured follow-up for at least a period of 5 years is mandatory. According to the German S3 guidelines, follow-up includes anamnesis, physical examination, ultrasound of the liver, chest x-ray, rectoscopy, and colonoscopy. The estimation of carcinoembryonic antigen (CEA) preoperatively and every 6 months for at least 2 years during follow-up is recommended for early detection of recurrence of disease, followed by further systemic or surgical therapy [5]. Unfortunately, at the time of diagnosis of CRC, CEA is only in 39–50% of the cases elevated and therefore only of limited use for monitoring of follow-up [37]. Of note, also in the cohort included in this study, CEA was elevated only in 33% of the patients.

In agreement with the literature, our own work suggested that ICAM-1 is a biomarker associated with cancer progression. In previous studies, we could demonstrate a significantly higher expression of ICAM-1 in tumor-associated fibroblasts as compared to normal tissue-associated fibroblasts [19]. Elevated concentrations of sICAM-1 have been described in a relevant number of other cancers [21, 28–31]. Therefore, in the present study, the prognostic relevance of sICAM-1 concentrations in the sera of patients prior to operation was determined. First of all, we defined a cutoff value to differentiate high and low sICAM-1 concentrations according to a minimum *p* value approach. Such an approach identifies data-adaptively a cutoff value optimized to discriminate two subgroups with respect to their survival prognosis. In previous reports, sICAM-1 serum concentrations of healthy individuals were reported from 100 to 408 ng/ml [33]. Also, our control group presented with sICAM-1 concentrations between 149 and 530 ng/ml. A significant difference was found in the 5-year overall and cancer-related survival in patients with sICAM-1 concentrations below 290 ng/ml (sICAM-1^low^) compared to patients with sICAM-1 concentrations > 290 ng/ml (sICAM-1^high^). Moreover, a significant increase of sICAM-1 concentrations with progressing UICC stages, with the restriction combining the non-metastatic UICC stages I and II (*p* = 0.005), confirmed increased sICAM-1 concentrations in patients with locoregional or distant metastases compared to patients with localized disease. Importantly, increased levels of sICAM-1 were identified as independent prognostic factor with an increased hazard ratio of 1.6 in multivariate analyses.

The combination of CEA and sICAM-1 allowed an improved categorization of overall survival. For all four risk groups, we found a significant difference in overall survival (*p* < 0.001) and cancer-related survival (*p* < 0.001). Patients with elevated CEA concentrations as well as sICAM-1^high^ concentrations presented with the worst prognosis (overall 5-year-survival rate 29.1%; cancer-related survival rate 42.4%), whereas patients with normal CEA concentrations and sICAM-1^low^ concentrations presented with the best prognosis of all patients (overall 5-year-survival rate 85.4%; cancer-related survival rate 92.2%). Patients with normal CEA and elevated sICAM-1 presented with a better outcome (overall 5-year survival rate 72.6%; cancer-related survival rate 86.0%) than patients with elevated CEA and sICAM-1^low^ (overall 5-year survival rate 48.7%; cancer-related survival rate 59.1%). Importantly, the higher risk groups (either sICAM-1^high^ or combined score 3 or 4) were not comprised of stage IV patients only. Instead, all score categories included patients of all four UICC stages and the relative proportion of lower UICC stages decreased with progressing risk score (Fig. 2c). The specific benefit of the study is the large size of our study group (*n* = 297) and the long follow-up period with a median of 66 months.

## Conclusions

CEA is a well-known marker for the estimation of further prognosis in CRC. However, frequently, this marker is not elevated at the time of operation and therefore less useful for assessment of prognosis in these patients. In such cases, sICAM-1 adds further prognostic value and enables the identification of high-risk subgroups in patients with either normal or elevated CEA concentrations. Soluble ICAM-1 is an inflammation-associated marker and is therefore increased in patients with an inflammatory tumor microenvironment. An UICC stage–dependent follow-up is mandatory in CRC, but in patients with sICAM-1 concentrations ≥ 290 ng/ml, special carefulness or even a tighter follow-up is recommended. Furthermore, it may be discussed whether patients with UICC stage II colon cancer and a high-risk CEA/sICAM-1 score might profit from adjuvant chemotherapy. Moreover, soluble ICAM-1 is easily measurable by commercially available ELISAs and can therefore readily be integrated in clinical routine.
